# MicroRNA-29b-2-5p inhibits cell proliferation by directly targeting Cbl-b in pancreatic ductal adenocarcinoma

**DOI:** 10.1186/s12885-018-4526-z

**Published:** 2018-06-25

**Authors:** Ce Li, Qian Dong, Xiaofang Che, Ling Xu, Zhi Li, Yibo Fan, Kezuo Hou, Shuo Wang, Jinglei Qu, Lu Xu, Ti Wen, Xianghong Yang, Xiujuan Qu, Yunpeng Liu

**Affiliations:** 1 Department of Medical Oncology, the First Hospital of China Medical University, NO.155, North Nanjing Street, Heping District, Shenyang City, 110001 China; 2 Key Laboratory of Anticancer Drugs and Biotherapy of Liaoning Province, the First Hospital of China Medical University, Shenyang, 110001 China; 3 Department of Oncology, Shengjing Hospital of China Medical University, Shenyang, 110004 China; 4 Department of Pathology, Shengjing Hospital of China Medical University, Shenyang, 110004 China

**Keywords:** PDAC, Prognosis, miR-29b-2-5p, Cbl-b, p53, Proliferation

## Abstract

**Background:**

MicroRNAs can be used in the prognosis of malignancies; however, their regulatory mechanisms are unknown, especially in pancreatic ductal adenocarcinoma (PDAC).

**Methods:**

In 120 PDAC specimens, miRNA levels were assessed by quantitative real time polymerase chain reaction (qRT-PCR). Then, the role of miR-29b-2-5p in cell proliferation was evaluated both in vitro (Trypan blue staining and cell cycle analysis in the two PDAC cell lines SW1990 and Capan-2) and in vivo using a xenograft mouse model. Next, bioinformatics methods, a luciferase reporter assay, Western blot, and immunohistochemistry (IHC) were applied to assess the biological effects of Cbl-b inhibition by miR-29b-2-5p. Moreover, the relationship between Cbl-b and p53 was evaluated by immunoprecipitation (IP), Western blot, and immunofluorescence.

**Results:**

From the 120 PDAC patients who underwent surgical resection, ten patients with longest survival and ten with shortest survival were selected. We found that high miR-29b-2-5p expression was associated with good prognosis (*p* = 0.02). The validation cohort confirmed miR-29b-2-5p as an independent prognostic factor in PDAC (*n* = 100, 95% CI = 0.305–0.756, *p* = 0.002). Furthermore, miR-29b-2-5p inhibited cell proliferation, induced cell cycle arrest, and promoted apoptosis both in vivo and in vitro. Interestingly, miR-29b-2-5p directly bound the Cbl-b gene, down-regulating its expression and reducing Cbl-b-mediated degradation of p53. Meanwhile, miR-29b-2-5p expression was negatively correlated with Cbl-b in PDAC tissues (*r* = − 0.33, *p* = 0.001).

**Conclusions:**

Taken together, these findings indicated that miR-29b-2-5p improves prognosis in PDAC by targeting Cbl-b to promote p53 expression, and would constitute an important prognostic factor in PDAC.

**Electronic supplementary material:**

The online version of this article (10.1186/s12885-018-4526-z) contains supplementary material, which is available to authorized users.

## Background

Pancreatic ductal adenocarcinoma (PDAC) is one of the most lethal solid tumors, with an exceedingly poor prognosis [1]. Despite great achievements in surgery, chemotherapy and radiotherapy, the 5-year survival rate of patients with PDAC remains low, less than 7% [2]. One of the reasons underlying poor prognosis in pancreatic cancer is that pancreatic cancer cells have a very strong proliferative capacity [3]. A wide range of prognostic factors are associated with proliferation, including vascular endothelial growth factor (VEGF) [4, 5], insulin-like growth factor(IGF) [6], nerve growth factor receptors (NGF) [7], transforming growth factor (TGF)-β [8]; however, their roles in PDAC have been assessed at the protein level. Increasingly, genetic and epigenetic, more recently, microRNA alterations are found in multiple tumors [9–11]. However, how miRNAs affect tumor progression or patient outcome is unclear, especially in PDAC.

MicroRNAs (miRNAs) are non-coding small RNAs, with a length of 20–23 nucleotides [12]. They bind specific target mRNAs in the 3′-untranslated region (UTR), resulting in target mRNA degradation or translation inhibition, which may affect cell proliferation [13]. Due to high stability, small size, tissue specificity and simple isolation, miRNAs are more advisable as prognostic predictive biomarkers than mRNAs and proteins. Accumulating evidence strongly suggests that aberrant miRNA expression is a common and important feature of human malignancies, facilitating proliferation and promoting prognosis [14–17]. The expression levels of several miRNAs, including miR-125b, miR-199a, miR-100, let-7 g, miR-433 and miR-214, are associated with the progression and prognosis of gastric cancer [18]. A serum miRNA classifier (miR-21-5p, miR-20a-5p, miR-103a-3p, miR-106b-5p, miR-143-5p, and miR-215) is considered a stable prognostic tool for detecting disease recurrence in patients with stage II colon cancer [19]. However, studies assessing the prognostic significance of miRNAs in PDAC are scarce.

As an essential enzyme in the ubiquitin-proteasome system (UPS), Casitas B-lineage lymphoma (Cbl)-b functions as E3 ubiquitin ligase or multifunctional adaptor protein [20, 21]. In previous studies on solid tumors, Cbl-b is mostly focused on gastric cancer [22], breast cancer [23], and non-small-cell lung cells [24]. The function in those solid tumors are inhibiting the proliferation. But the relationship between Cbl-b and PDAC is less reported [25, 26]. We previously studies showed that silencing Cbl-b expression activated the Smad3/p21 axis and inhibited proliferation of PDAC cells [25]. However, the relationship between miRNA and Cbl-b as well as the Cbl-b related protein in PDAC is unclear. Whether Cbl-b plays a role in the prognosis of miRNA-expressing PDAC patients remains to be elucidated. Interfering with miRNA-Cbl-b expression or miRNA-Cbl-b signaling pathway may prolong the survival rate of PDAC patients, thereby elucidating potential therapeutic targets and prognostic biomarkers.

The present study demonstrated that miR-29b-2-5p was a good independent prognostic factor in resectable pancreatic cancer. Furthermore, miR-29b-2-5p negatively regulates Cbl-b to reduce Cbl-b-mediated ubiquitination and p53 expression, inhibiting the proliferation of PDAC cells.

## Materials

### Human tissue samples

Freshly isolated human PDAC tissues from 120 patients and adjacent pancreatic tissues were obtained with informed consent from the Department of Pathology, the affiliated Shengjing Hospital, China Medical University, between January 2009 to Feburary 2011. The clinic-pathologic characteristics and prognosis were available for 120 patients. The patients had not received chemotherapy or radiation therapy prior to surgery.

Each case diagnosis and histological grade, there are two pathologists confirmed based on the American joint committee on pathological diagnosis. Patient information included age, gender, location of tumor, Maximum tumor diameter, differentiation, surgical margins, pT category, pN category, vessel invasion, vascular tumor thrombus, adjacent organs invasion, pTNM category and Overall survival(OS). The maximal tumor size was defined as the maximum diameter on pathologic analysis. The tumor was staged according to the American Cancer Association (TNM’s AJCC staging system) 2010. The final survival data were collected in 31 December 2014. During the 120 cases, 20 cases were analyzed with miRNA microarray. Because they were similar in clinic-pathologic features and treatment but were different in outcomes. The medium OS used as cut off value reference to previous studies [27, 28]. Half of the patients died within the first year of diagnosis were classified as “poor prognosis” with median OS of 6.3 months. Patients who survived more than 21 months had a median OS of 48.0 months, which classified as the “good prognosis” group. The background of the clinic-pathologic characteristics of the 20 patients has been published on our previous study [25]. This study was approved by the Human Ethics Review Committee of China Medical University (protocol #: 2015PS63K); informed consent was obtained from all patients in accordance.

### Cell lines and culture conditions

The human pancreatic adenocarcinoma cell lines SW1990(#TCHu201), Capan-2(#SUER0449) were obtained from the Type Culture Collection of the Chinese Academy of Sciences (Shanghai, China) and Suer Biological Technology(Shanghai, China) respectively. Before the experiments, the two cell lines were authenticated on cell micrograph compared to the cell lines on ATCC. The cell lines were maintained in RPMI 1640 medium that contained 10% heat-inactivated foetal bovine serum (FBS), penicillin (100 U/ml) and streptomycin (100 mg/ml) under 5% CO2 at 37 °C.

### Transient transfection

MiR-29b-2-5p mimic and the negative control were obtained from RiboBio (Guangzhou, China). p3XFLAG—CMV9(NC) and p3XFLAG—CMV9 Cbl-b (OE Cbl-b) were obtained from Sigma(USA). The small interfering RNA sequences (Genepharma, Shanghai, China) for Cbl-b was 5′-CCUGAUGGGAGGAGUUAUAtt-3′ (sense), 5′-UAUAACUCCUCCCAUCAGGtt − 3′ (antisense).MiRNAs and siRNAs transfection was performed using Lipofectamine 2000 (Invitrogen) according to the manufacturer’s instruction.

### MicroRNA microarray

The levels of total human microRNAs’ expression were quantified using a GenoSensor’s GenoExplorerTM microRNA microarray (Tempe, AZ, USA). The hybridized miRNA chips were scanned and analyzed using an Axon GenePix 4000B scanner and GenePix Pro software (Molecular Devices, CA, USA).

### RNA extraction and quantitative reverse transcription real-time polymerase chain reaction (qRT-PCR)

Total RNA extracted as described above [25]. For miRNA detection, reverse transcription was performed using One Step PrimeScript® miRNA cDNA Synthesis kit (Takara, Japan), and real-time polymerase chain reaction (PCR) was carried out using SYBR® premix Ex Taq™ II (TaKaRa, Japan) with the ABI 7500 Sequence Detection System (Applied Biosystems, Foster, CA). The sequences (TaKaRa, Japan) for miR-29b-2-5p was 5′-CCTTCGACATGGTGGCTTAGAAA-3′, and U6 was 5′-GCTTCGGCAGCACATATACTAAAAT-3′(sense) and 5′-CGCTTCACGAATTTGCGTGTCAT-3′(anti-sense). The PCR conditions were 30 s at 95 °C, followed by 45 cycles at 95 °C for 5 s, and 58 °C for 25 s. Data were analyzed using the Applied Biosystems 7500 software program (version 2.3) with the automatic Ct setting for adapting baseline and threshold for Ct determination. The threshold cycle and 2-^ΔΔCt^ method were used for calculating the relative amount of the target RNA.

### Reverse-transcription-polymerase chain reaction (RT-PCR)

For mRNA detection, reverse transcription was performed using the M-MLV Reverse Transcriptase System (Promega, USA). RT-PCR was performed with the following primer pairs for Cbl-b: forward (5′-CGCTTGACATCACTGAAGGA-3′); and reverse (5′-CTTGCCACACTCTGTGCATT-3′). GAPDH was used as a control: forward (5′-GTGGGGCGCCCCAGGCACCA-3′); and reverse (5′-CTCCTTAATGTCACGCACGATTTC-3′). PCR conditions for Cbl-b were 95 °C for 5 min, 30 cycles at 95 °C for 30 s, 59 °C for 30 s, 72 °C for 30 s, and 1 cycle at 72 °C for 10 min. GAPDH were 95 °C for 5 min, 33 cycles at 95 °C for 30 s, 56 °C for 45 s, 72 °C for 45 s, and 1 cycle at 72 °C for 10 min. The amplified products were separated on 1% agarose gels, and stained with ethidium bromide and visualized under UV illumination.

### Cell proliferation assay

To evaluate the effects of miR-29b-2-5p on cell growth, SW1990 and Capan-2 PDAC cells were incubated in the 6-well plates (3 × 10^5^ cells per hole) in triplicate. The next day, the cells were transfected with miR-29b-2-5p mimics or negative control mimics (NC; Ribobio, China) or OE Cbl-b/NC(1.5 μg) using Lipofectamine 2000 (Invitrogen). The final concentration was kept constant (50 nmol/L). Measure the culture of cell proliferation, cell in 2 ml medium, counted manually after 24, 48, 72, and 96 h use the hemacytometer (Hawksley, West Sussex, UK) and bright field microscope. It combined with Trypan blue staining method to determine growth state of dispersed cells.

### Dual luciferase reporter assay

The 3′-UTR sequence of Cbl-b was obtained through gene synthesis (OriGene, Rockville, MD, USA), and then cloned into the vector pMirTarget through two restriction enzyme cutting sites (SgfI-MluI), resulting in the generation of SC209114. The reagents and methods are provided by OriGene Technologies (OriGene, Rockville, MD, USA). And the sequencing results were compared with the standard template sequences of the BLAST software on the PUBMED and CHROMAS software to identify the gene mutation *loci*. To generate the Cbl-b mutant reporter, the seed region was mutated to remove all complementary nucleotides to miR-29b-2-5p. PDAC cells were co-transfected with firefly luciferase reporter plasmids(0.5 μg), pRL-TK luciferase control vector(0.005 μg) and miR-29b-2-5p or NC(50 nmol) in the 24-well plates. Luciferase assays were performed 24 h after transfection, using the dual-luciferase reporter assay system (Promega, Madison, WI, USA) according to the manufacturer’s protocol.

### Western blotting analysis

Western blotting was performed as our previously described [29]. The primary antibodies, anti-Cbl-b, anti-b-actin, anti-p53, anti-Bax-2, anti-Bcl-1, anti-GAPDH, anti-UB were from Santa Cruz Biotechnology (Santa Cruz, CA); anti-IgG was from Cell Signaling Technology (Beverly, MA). Enhanced chemiluminescence reagent (SuperSignal Western Pico Chemiluminescent Substrate; Pierce, USA) were used to analysis proteins. The final result was analyzed by NIH Image J software.

### Cell cycle analysis

Cells were fixed with 70% ice-cold ethanol overnight. Fixed cells were resuspended in PBS containing 10 μg/ml propidium iodide (PI, KeyGEN, China), 0.1% Triton, and 20 μg/ml RNase A (KeyGEN) and were incubated for 30 min in the dark. Finally, the samples were evaluated by flow cytometry and the data were analyzed with Flow Cytometry (BD Accuri C6; BD Biosciences, San Jose, CA, USA) and analyzed with WinMDI version 2.9 software (The Scripps Research Institute, La Jolla, CA, USA).

### Cell apoptosis assay

Transfected cells were cultured in six-well plates. Samples were subsequently stained using an Annexin V-fluorescein isothiocyanate/propidium iodide apoptosis detection kit (cat no. BMS500FI-100; Invitrogen; Thermo Fisher Scientific, Inc.) and the number of apoptotic cells was determined by FACS Calibur flow cytometry (BD Biosciences, San Jose, CA, USA), according to the manufacturer’s protocol. Finally, the results were analyzed with WinMDI v.2.9 software (The Scripps Research Institute, La Jolla, CA, USA).

### In vivo tumor growth model

All in vivo studies were approved by the Institutional Review Board of China Medical University. These animals were cared of in accordance with institutional ethical guidelines of animal care. Female SPF BALB/c nude mice were bought from Vitalriver (Beijing, China). Mice were sacrificed in gas chamber and by cervical dislocation to confirm death according to the protocol filed with the Guidance of Institutional Animal Care and Use Committee of China Medical University. SW1990 cells (1 × 10^6^) with 0.15 ml PBS subcutaneous injected into mice’s right shoulder area. A week after the cells injected, randomly divided into two groups, each group of three mice, and mir-29-2b* agomir or mir-NC agomir (40 ul saline 5 nmol/L, Ribobio technology, Guangzhou, China) treatment by subcutaneous injection every 2 days. Every 2 days with a caliper measuring the volume of tumor, the calculation of tumor volume, use the following formula: V = 1/2 (width×length×height).Body weights were also recorded. With the protocol to the Animal Care and Use Ethnic Committee the China Medical University under the protocol number 16080 M, the tumor-bearing mice were sacrificed by cervical dislocation when the mice became moribund or on day 15.

### Immunoprecipitation(IP)

SW1990 cells were seeded at 3 × 10^5^ per well in six-well plates and incubated overnight; Cells were transfected with NC (1.5 μg), OE Cbl-b (1.5 μg) 24 h every six wells. The next day, the cells with OE Cbl-b treated with or without proteasome inhibitor PS341 (5 nM) for 24 h. After removal of the medium, cells were transferred to 1.5 ml EP tube for transient centrifugalization. Cell pellets were washed by ice-cold PBS for two times. For immunoprecipitation, cells were collected with denaturation buffer to separate protein complexes. Cell lysates were incubated with p53 antibody or immunoglobulin-G (1–4 μg, Cell Signaling Technology, MA) at 4 °C overnight followed by the addition of 20 μl of protein G-Sepharose beads (Santa Cruz Biotechnology) for an additional 2 h at 4 °C. The immunoprecipitated proteins with 3 × sampling buffer were eluted by heat treatment at 100 °C for 5 min.

### Immunofluorescence staining

Pancreatic cancer cells grew on Lab-Tek chamber slides (Nunc S/A, Polylabo, France). The following day, miR-29b-2-5p or NC (50 nmol/L) treated into cells for 48 h, 3.3% paraformaldehyde fixed for 15 min, 0.2% Triton X-100 permeabilized for 5 min, 5% bovine serum albumin (BSA) blocked for 1 h. And the cells incubated with anti-Cbl-b and anti-p53 antibody (Santa Cruz, CA) at a dilution of 1:200 overnight at 4 °C. Blocking solution for 1 h at room temperature with Alexa Fluor 546-conjugated goat anti-mouse IgG and Alexa Fluor 488-conjugated goat anti-rabbit IgG (Molecular Probes) in the dark. Nuclei was stained by 4′-6-diamidino-2-phenylindole for 5 min. The cells were visualized by fluorescence microscopy (BX53, Olympus, Japan).

### Immunohistochemistry(IHC)

One hundred of formalin-fixed, paraffin-embedded PDAC tissues were used for IHC. All sections were performed using the following antibodies: anti-Cbl-b (Santa Cruz Biotechnology) using S-P immunohistochemical kit (Fuzhou Maixin Biological Technology Ltd., Fujian, China) as described previously [30]. The scanning the entire tissue specimen evaluated the staining under low magnification (× 10) and confirmed under high magnification (× 20 and × 40). Visualized and classified the protein expression was based on the percentage of positive cells and the intensity of staining. Tumors with < 10% Cbl-b expression were regarded as negative or weak (0),10–70% were regarded as moderate (1) and ≥ 70% were considered positive (2). The cut off of weak-medium-strong is 10 and 70% respectively. Final scores were assigned by two independent pathologists.

### Statistical analysis

Statistical analysis was performed using the GraphPad Prism software (La Jolla, CA, USA). Overall survival (OS) was defined as the time from the date of the surgery to the date of death or the last contact, i.e., the date of the last follow-up visit. Kaplan-Meier estimate was used to analyze the survival data and the statistical significance was evaluated by the log rank test. ROC curve from the point to cut off value is based on the previously study [31]. Multivariate analysis was performed using the multivariate Cox proportional hazards model (forward), which was fitted using all of the clinic-pathologic variables. Chi-square test was used to evaluated the correlation between miR-29b-2-5p expression levels and the clinical characteristics. The differences between groups were assessed by Student’s t-test or Mann-Whitney U test. For correlation analysis, the non-parametric Spearman r tests were applied. All means were calculated from at least three independent experiments. Two-sided *P* values < 0.05 were considered to be statistically significant. SPSS software (version 13.0; SPSS, Inc. Chicago, IL, USA) was used for statistical analysis.

## Results

### MiR-29b-2-5p is correlated with good prognosis in pancreatic cancer

**Fig. 1:**
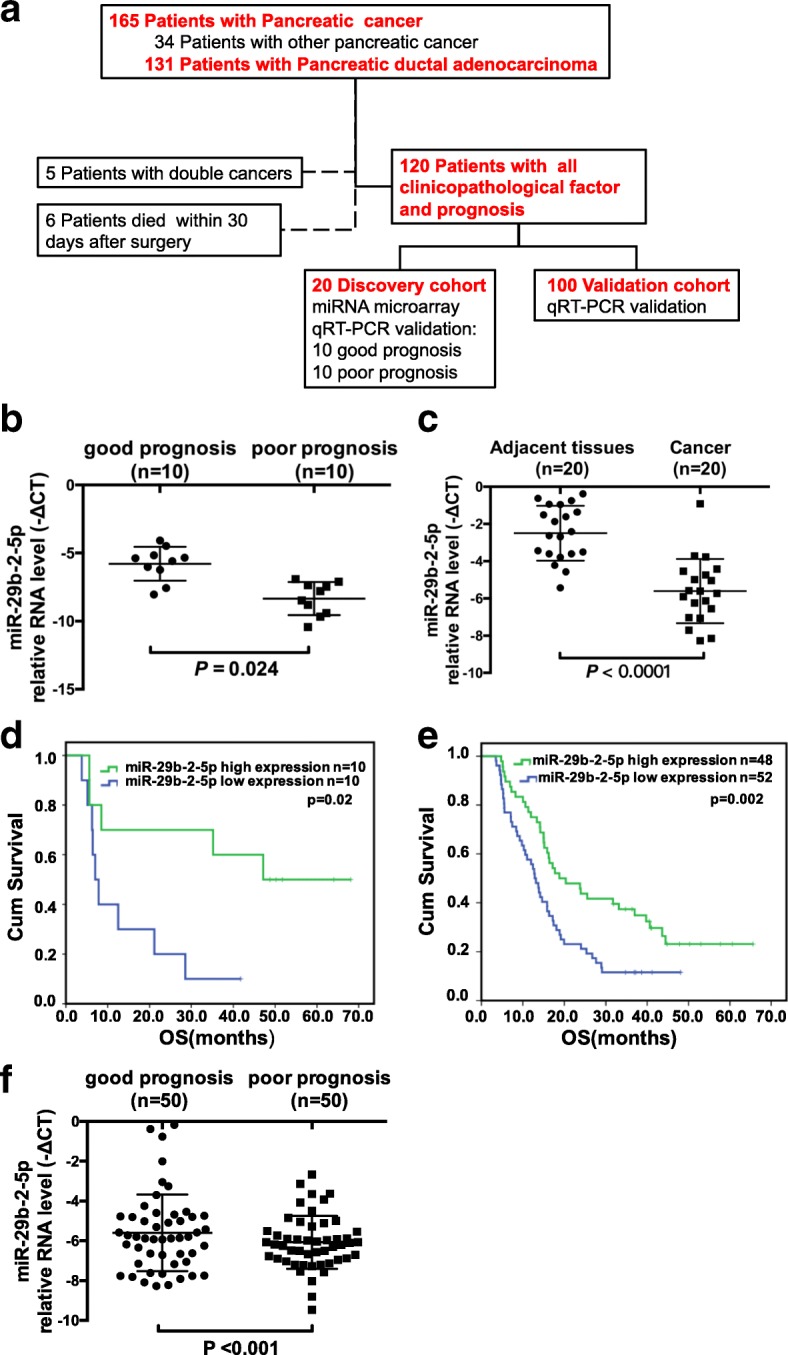
miR-29b-2-5p has a positive correlation with the prognosis of pancreatic cancer and independently predicted better survival. **a** The flowchart of patient selection and schematic design. **b** Statistical analysis of miR-29b-2-5p expression in good and poor prognosis group, nonparametric Mann–Whitney test. All the bars represent SE. **c** Statistical analysis of miR-29b-2-5p expression in normal and cancerous pancreatic tissues, nonparametric Mann–Whitney test. All the bars represent SE. **d** In miRNA array cohort, miR-29b-2-5p high expression associated with a median survival of 35.2 months versus low expression of 6.4 months (log rank x^2^ = 21.837, *p* = 0.02). **e** In miRNA validation cohort, patients with high or low miR-29b-2-5p expression associated with a median OS respectively time of 18.8 or 12.9 months. (log rank x^2^ = 9.296, *p* = 0.002). **f** The good prognosis group levels of miR-29b-2-5p in these 100 validation cohort is higher than poor prognosis group. (*p* < 0.001)

**Fig. 2:**
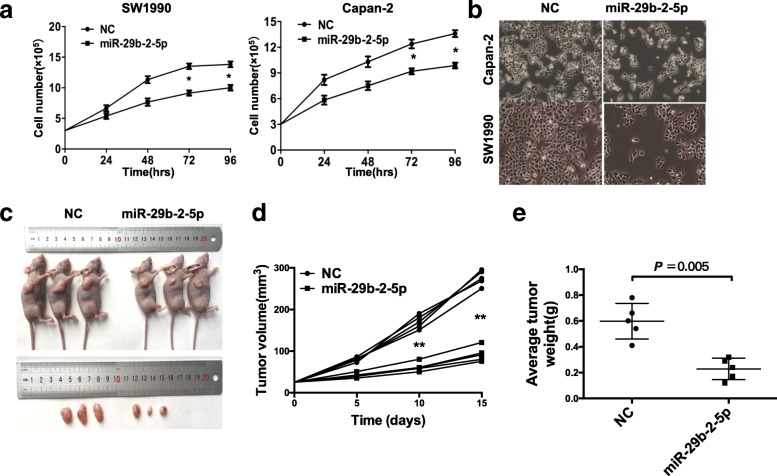
miR-29b-2-5p inhibits PDAC cell proliferation in vitro and in vivo experiments systems. **a** PDAC cell lines, SW1990 and Capan-2, were transfected with miR-29b-2-5p or NC. Cells were collected at 48, 24, 72, and 96 h after transfection using Trypan blue staining method. The results suggested miR-29b-2-5p significantly inhibited the proliferation of PDAC cells (mean ± SD, results of three independent experiments, *P < 0.05). **b** Observation under microscope of the cells transfected with miR-29b-2-5p or NC 72 h after transfection. The number of cells in miR-29b-2-5p group was significantly decreased compared with that in NC group. **c** miR-29b-2-5p agomir was intratumorally injected after the tumor was formed. After 2 weeks, the size of the subcutaneous tumor treated with miR-29b-2-5p agomir significantly decreased compared with NC-treated tumor. **d** Quantification of tumor volume development in NC- and miR-29b-2-5p-bearing nude mice. **e** Subcutaneous tumors derived from SW1990 cells in the NC- or miR-29b-2-5p agomir-treated group were weighed after tumors were harvested in histogram, *P < 0.05, **P < 0.001

The flowchart of patient selection and schematic design were shown in Fig. 1a. We performed a comprehensive microarray analysis to compare miRNA expression profiles in pancreatic tissues from two groups of participants. Our previous study showed that patients with good prognosis, median OS was 48.0 months, compared to 6.3 months in those with poor prognosis. There was no statistically significant differences in the remaining clinical and pathological features between the two groups, corroborating previous findings [25]. The good prognosis group had 22 miRNAs significantly upregulated (miR-29b-2-5p, etc.) as demonstrated by miRNA microarray analysis [25]. Among these candidate miRNAs, 4 miRNAs are Dead miRNA Entry through miRbase which we cannot get the sequences. We used real-time PCR to test the result of miRNA array. In the rest of 18 candidate miRNAs, 2 miRNAs were opposite from the miRNA array, 16 were coherent with the miRNA array (see Additional file 1: Figure S2.A.B online). We tried to find targets which can be regulated by the miRNAs, and found 7 miRNAs had targets with softwares miRwalk and starBase. Among these candidate 7 miRNAs, miR-29b-5p, miR-891b and miR-490-5p could inhibit proliferation in cell lines, and miR-29b-2-5p was most stable in inhibiting PDAC tumor cell proliferation as well as the result of microarray (see Additional file 1: Figure S2.C online, Fig. 2a). Real-time PCR confirmed that miR-29b-2-5p was associated with better prognosis. MiR-29b-2-5p expression gradually increased from the poor to good prognosis groups (Fig. 1b), and from cancer to adjacent pancreatic tissues (Fig. 1c). Furthermore, high miR-29b-2-5p expression was associated with a median OS of 35.2 months versus 6.4 months for the low expression group (log rank x^2^ = 21.837, *p* = 0.02; Fig. 1d). A strong correlation between miR-29b-2-5p expression status and OS was demonstrated, confirming that miR-29b-2-5p was a prognostic factor in PDAC.

To verify the prognostic role of miR-29b-2-5p, the expression levels of this miRNA were assessed by qRT-PCR in 100 independent PDAC samples. This validation cohort contained stage I, II and III tumors. Other clinical pathologic features were not significantly different from those of the initial patient cohort (see Additional file 2: Table S1). We also evaluated the correlation between miR-29b-2-5p expression levels and the clinical characteristics using chi-square test (Table 1), found that Gender (*p* = 0.028), Maximum tumor diameter (cm) (*p* = 0.11), Differentiation (*p* < 0.001), Surgical margins (*p* < 0.001), pT category (*p* = 0.002), pN category (*p* < 0.001), Vascular tumor thrombus (*p* < 0.001), Adjacent organs invasion (*p* < 0.001), CA19–9((*p* < 0.001) had correlation with miR-29b-2-5p. MiR-29b-2-5p was detected in all patients. Patients with high miR-29b-2-5p expression had median OS of 18.8 months (95% CI 10.4–27.3 months) versus 12.9 months (95% CI 10.6–15.1 months) for the low expression group (log rank χ^2^ = 9.296, *p* = 0.002; Fig. 1e). And scatter plot showed that the good prognosis group levels of miR-29b-2-5p in these 100 validation cohort is higher than poor prognosis group (*p* < 0.001, Fig. 1f). We also use ROC analyses based on clear cut-off values on which expression levels miRNA-29b-2-5p is prognostic relevant. The result is the same as Medium method. (see Additional file 3: Figure S1 online).

**Table 1:** The correlation between miR-29b-2-5p expression levels and the clinical characteristics

Characteristics	Cases	miR-29b-2-5p expression in PDAC		
		Low(%)	High(%)	*P* value
Age (years)				0.689
< 60	48	25(52.1)	23(47.9)	
≥ 60	52	27(51.9)	25(48.1)	
Gender				0.028*
Male	61	33(54.1)	28(45.9)	
Female	39	19(48.7)	20(51.3)	
Location of tumor				0.072
Head	59	27(45.8))	32(54.2)	
Body or tail	41	25(61)	16(39)	
Type of operation				0.088
Pancreaticoduodenectomy	77	43(55.8)	34(44.2)	
Distal pancreatectomy	23	9(39.1)	14(60.9)	
Total pancreatectomy	0	0	0	
Maximum tumor diameter (cm)				0.11
< 4	42	28(66.7)	14(33.3)	
≥ 4	58	24(41.4)	34(58.6)	
Differentiation				< 0.001*
Well	25	13(52)	12(48)	
Moderately	59	28(47.5)	31(52.5)	
Poor	16	11(68.8)	5(31.2)	
Surgical margins				< 0.001*
Negative	97	51(52.6)	46(47.4)	
Positive	3	1(33.3)	2(66.7)	
pT category				0.002*
pT1	11	7(63.6)	4(36.4)	
pT2	38	20(52.6)	18(47.4)	
pT3	24	12(50)	12(50)	
pT4	27	13(48.2)	14(51.8)	
pN category				< 0.001*
pN0	73	37(50.7)	36(49.3)	
pN1	27	15(55.6)	12(44.4)	
Vessel invasion				0.841
No	51	32(62.8)	19(37.2)	
Yes	49	20(40.8)	29(59.2)	
Vascular tumor thrombus				< 0.001*
No	97	49(50.5)	48(49.5)	
Yes	3	3(100)	0	
Adjacent organs invasion				< 0.001*
No	83	43(51.8)	40(48.2)	
Yes	17	9(52.9)	8(47.1)	
pTNM category				0.075
I	44	23(52.3)	21(47.7)	
II	29	15(51.7)	14(42.3)	
III	27	14(51.9)	13(48.1)	
CA19-9 (U/mL)				< 0.001*
≥ 37	87	45(51.7)	42(48.3)	
< 37	13	7(53.9)	6(46.1)	

*pT* pathologic T, *pN* pathologic N, *pTNM* pathologic TNM

*Values shown in bold italics are statistically significant

Multivariate Cox proportional hazard model (forward) was used to fit all 15 clinical pathological variables. MiR-29b-2-5p was included in the multivariate Cox proportional hazards model (forward) analysis of 100 patients along with prognostic clinic-pathologic factors. High miR-29b-2-5p expression (HR, 0.492; 95% CI, 0.300–0.807; *P* = 0.005), pT4 category (HR, 1.286; 95% CI 1.004–1.646; *P* = 0.046), serum CA19–9 level ≥ 37 U/ mL (HR, 3.47; 95% CI, 1.484–8.112; *P* = 0.004), and poorly differentiated tumor (HR, 1.472; 95% CI 1.016–2.133; *P* = 0.041) were significant independent prognostic factors associated with OS (Table 2). These data suggested that miR-29b-2-5p represented a tumor suppressor in PDAC.

**Table 2:** Multivariate Cox regression analysis including miR-29b-2-5p expression levels and overall survival in 100 patients with PDAC

Variables	Univariable analysis			Multivariable analysis		
	HR	95% CI	*P* value	HR	95% CI	*P* value
miR-29b-5p(high/low)	0.503	0.32–0.788	0.003	0.492	0.300–0.807	0.005
pT category(T4/T3/T2/T1)	1.212	0975–1.508	0.084	1.286	1.004–1.646	0.046
pN category(N1/N0)	1.871	1.147–3.053	0.012			
CA 19-9(≥ 37 U/mL/<37 U/mL)	3.315	1.426–7.706	0.005	3.47	1.484–8.112	0.004
Tumor Differenciation (Poor/Moderately/Well)	1.45	1.014–2.074	0.042	1.472	1.016–2.133	0.041

The multivariate Cox proportional hazards model (forward) was fitted using all of the clinical and pathological variables, which included age (≥60 vs. <60 years old), gender (male vs. female), type of operation (pancreaticoduodenectomy vs. distal pancreatectomy vs. total pancreatectomy), surgical margins (positive vs. negative), location of tumor (head vs. body or tail), maximal tumor diameter, histological differentiation (poorly vs. moderately vs. well differentiated), pT category (pT4 vs. pT3 vs. pT2 vs. pT1), pN category (pN1 vs. pN0), vessel invasion (yes vs. no), vascular tumor thrombus (yes vs. no), adjacent organs invasion (yes vs. no), pTNM category (I vs. II vs. III), miR-29b-2-5p expression (high expression vs. low expression), and CA19–9 level (≥37 U/mL vs. < 37 U/mL)

### MiR-29b-2-5p inhibits pancreatic cancer proliferation, and induces PDAC cell apoptosis and G1 phase cell cycle arrest

To assess whether miR-29b-2-5p plays a tumor suppressive role in PDAC development, we first evaluated the effect of miR-29b-2-5p on cell proliferation using the Trypan blue staining method in Capan-2 and SW1990 cells. MiR-29b-2-5p-treated Capan-2 and SW1990 cells exhibited significantly lower growth rates compared with control cells (Fig. 2a, b). Increased miR-29b-2-5p expression upon treatment of the two PDAC cell lines was confirmed by qRT-PCR (see Additional file 4: Figure S3 online). These results provided strong evidence that miR-29b-2-5p was a negative regulator of pancreatic cancer development and progression. To determine whether miR-29b-2-5p could have a potential therapeutic value in vivo, nude mice bearing subcutaneous SW1990 xenografts were treated with miR-29b-2-5p every other day for 14 days. After euthanasia, the tumors were removed from the animals for analysis (Fig. 2c–e). The results suggested that miR-29b-2-5p might have a therapeutic potential for the treatment of PDAC.

**Fig. 3:**
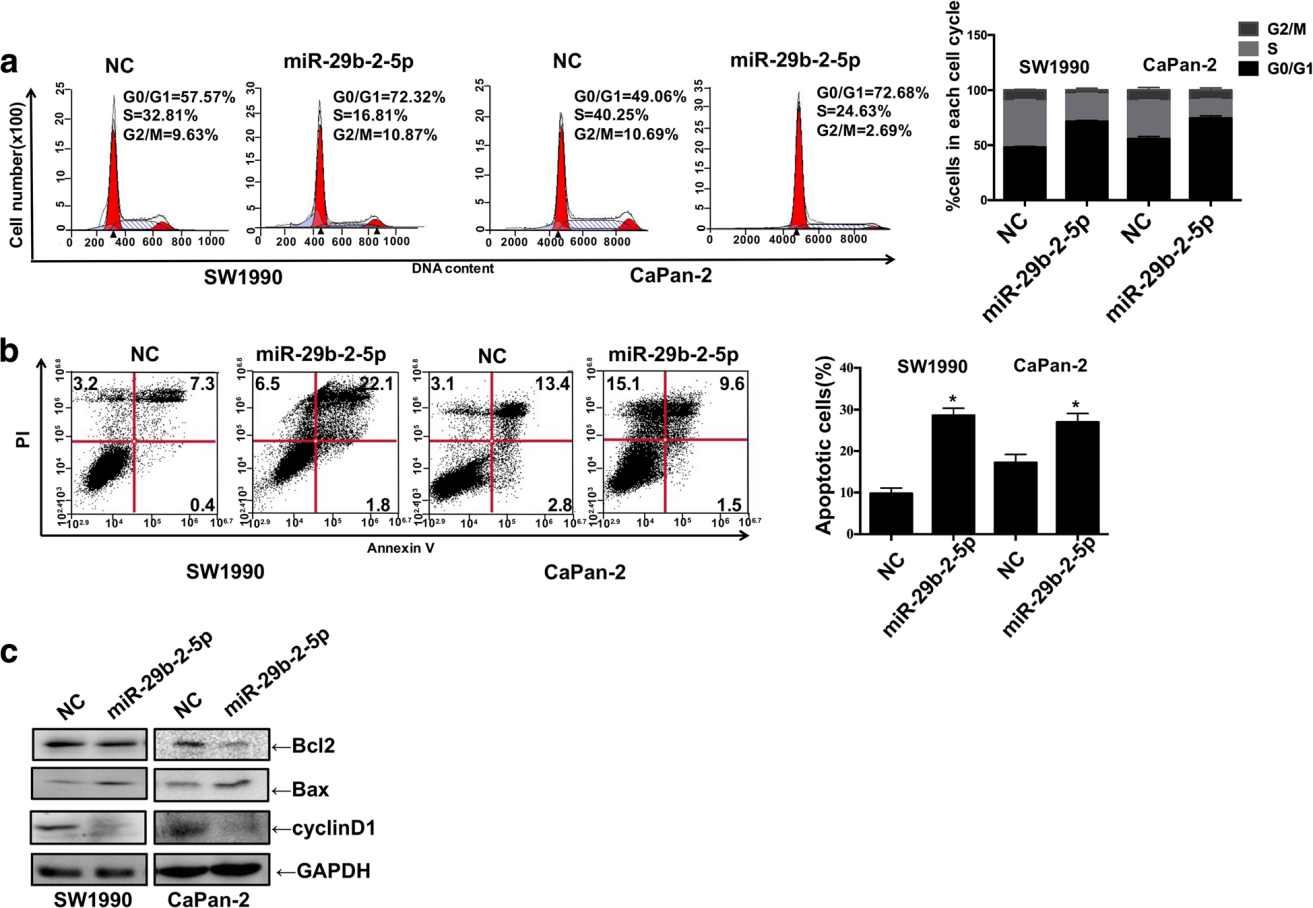
Upregulation of miR-29b-2-5p expression induces PDAC cells apoptosis and G1 phase cell cycle arrest. SW1990 and Capan-2 were transiently transfected with miR-29b-2-5p mimic. Forty-eight hours later, cell cycle arrest (**a**) and apoptosis (**b**) were analyzed by flow cytometry. The error line represents the mean ± SD, *P < 0.05. Forty-eight hours later, whole cell lysate was used for the Western blotting analysis. Cyclin D1, Bcl-2, Bax, and GAPDH were detected with their respective antibodies; *n* = 3 (**c**). Data are presented as mean ± SD (*n* = 3)

To further evaluate whether the miR-29b-2-5p-reduced cell proliferation was due to cell cycle arrest and/or apoptotic death, we first examined the effect of miR-29b-2-5p on cell cycle of SW1990 and Capan-2 cells. Compared with NC, the miR-29b-2-5p mimic significantly enhanced the G0/G1 subpopulation in SW1990 and Capan-2 cells (Fig. 3a). As shown in Fig. 3b, miR-29b-2-5p significantly promoted apoptosis in PDAC cells. In agreement, miR-29b-2-5p significantly reduced the levels of Bcl-2 and cyclinD1, and enhanced Bax2 amounts (Fig. 3c). These data suggested that miR-29b-2-5p up-regulation may promote cell cycle progression and inhibit cell apoptosis in PDAC cells.

### Cbl-b is a direct target of miR-29b-2-5p and involved in miR-29b-2-5p-induced tumor suppression

**Fig. 4:**
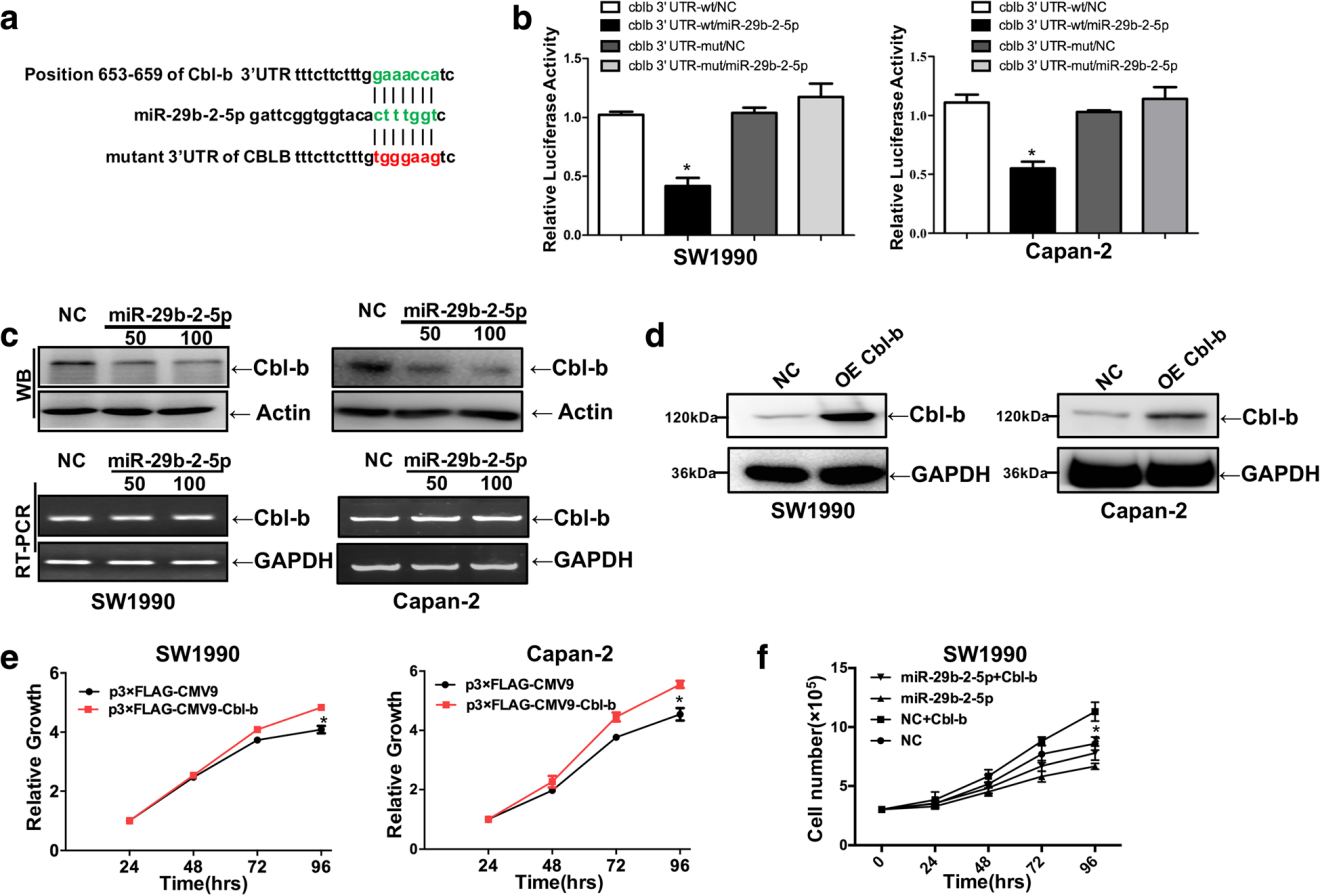
Cbl-b is a direct target of miR-29b-2-5p and involved in miR-29b-2-5p-induced tumor suppression. **a** Target site of miR-29b on 3UTRs of Cbl-b mRNA. The wild-type and mutated constructs were shown with the green and red seed region in bold. **b** Luciferase activity of pMirTarget-Cbl-b-wt or pMirTarget-Cbl-b-mut in Capan-2 and SW1990 cells after transfection with miR-29b-2-5por control. The error line represents the mean ± SD, *P < 0.05. **c** miR-29b-2-5p inhibited the expression of Cbl-b at the post-transcriptional level. SW1990 and Capan-2 were transfected with miR-29b-2-5p mimic in different concentrations. Western blot indicated miR-29b-2-5p down-regulated the expression of Cbl-b protein. RT-PCR suggested overexpression of miR-29b-2-5p did not significantly affect the level of Cbl-b mRNA; *n* = 3. **d** PDAC cell lines SW1990 and Capan-2 were transfected with p3xFLAG-CMV9-cbl-b (OE Cbl-b) or p3Xflag-CMV9(NC). Overexpression effect of Cbl-b was examined by Western blot; *n* = 3. **e** Cells were collected at 48, 24, 72, and 96 h after transfection using Trypan blue staining method. Take the 24 h/24 h, 48 h/24 h, 72 h/24 h, 96 h/24 h ratio respectively. The results suggested Cbl-b significantly promote the proliferation of PDAC cells (mean ± SD, results of three independent experiments, *P < 0.05). **f** SW1990 was co-transfected with a control nonspecific mimic (NC), miR-29b-2-5p, NC + p3xFLAG-CMV9-cbl-b and p3xFLAG-CMV9-cbl-b + miR-29b-2-5p. The results showed that miR-29b-2-5p could effectively reverse the effect of Cbl-b on the proliferation of PDAC cells

We used predicted softwares to screen the target gene of miR-29b-5p. In the top three candidate genes, Cbl-b changed most significantly. Our previous study reported that Cbl-b plays an important role in PDAC. Silencing of Cbl-b expression inhibited proliferation in PDAC cells [25]. In this work, the relationship between miR-29b-2-5p and Cbl-b was assessed. As shown in Fig. 4a, the miRNA/mRNA comparative analysis showed that the 3′UTR of Cbl-b had the binding site for miR-29b-2-5p, at 611–617 nt. To assess whether Cbl-b is regulated by miR-29b-2-5p through direct binding to its 3′UTR, we structured plasmids containing WT or mutant 3′UTR of human Cbl-b fused downstream of the firefly luciferase gene. WT and mutant plasmids were co-transfected into Capan-2 or SW1990 cells, respectively, with miR-29b-2-5p mimic or miR-NC. As shown in Fig. 4b, luciferase activity upon miR-29b-2-5p transfection was significantly reduced. Mutations of the Cbl-b 3′-UTR abrogated the suppressive effect of miR-29b-2-5p. RT-PCR showed that Cbl-b mRNA levels had no changes after miR-29b-2-5p treatment of both Capan-2 and SW1990 cells; miR-29b-2-5p repressed Cbl-b expression through post-transcriptional inhibition in human PDAC cells (Fig. 4c). These results suggested that Cbl-b serves as an actual target of miR-29b-2-5p.

To evaluate the effect of Cbl-b in PDAC cells, the overexpression plasmid targeting Cbl-b p3xFLAG-CMV9-cbl-b (OE Cbl-b) and control plasmid (NC) were transfected into SW1990 and Capan-2 cells. Cells with more than 50% of endogenous Cbl-b expression were used in subsequent experiments (Fig. 4d). The effect of Cbl-b on cell proliferation was assessed by the Trypan blue staining method. The results showed that Cbl-b could promote the proliferation of PDAC cells (Fig. 4e). To determine the impact of miR-29b-2-5p expression on PDAC biology, the levels of this miRNA in SW1990 cells were assessed after transfection with NC and miR-29b expression -2-5p, NC plus Cbl-b, or miR-29b-2-5p plus OE Cbl-b. The results showed that miR-29b-2-5p could effectively reverse the effect of Cbl-b on the proliferation of PDAC cells. (Fig. 4f).

### MiR-29b-2-5p promotes p53 expression by suppressing Cbl-b, likely through ubiquitination-dependent proteasomal degradation of p53

**Fig. 5:**
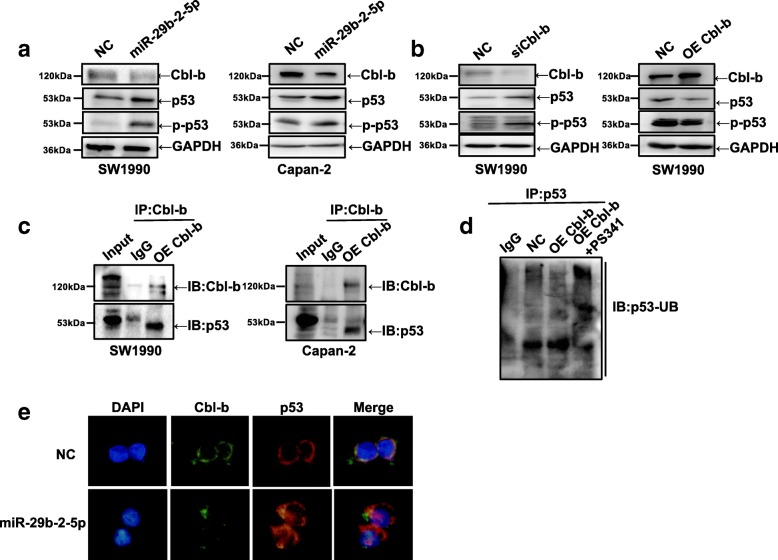
MiR-29b-2-5p can promote cell p53 by suppressing Cbl-b, and Cbl-b can ubiquitination-dependent proteasomal degradation of p53. **a** SW1990 and Capan-2 cells were transfected with miR-29b-2-5p 48 h. Cell lysates were collected for Western blot analysis by p53 and p-p53; *n* = 3. **b** SW1990 cells were transfected with siCbl-b or OE Cbl-b 48 h. Cell lysates were collected for Western blot analysis by p53 and p-p53; *n* = 3. **c** The interaction of p53 with Cbl-b was analyzed by coimmunoprecipitation. **d** The OE Cbl-b cells were treated with PS341 for indicated times. p53 was immunoprecipitated and ubiquitin was analyzed by western blot. OE Cbl-b, Cbl-b plasmid transfected; NC, no expression plasmid controls; *n* = 3. **e** SW1990 cells were treated with 50 nmol/L miR-29b-2-5p or NC miRNA for 48 h. Protein localization in the cells was assessed using immunofluorescent staining. Cbl-b (green) and p53 (red), DAPI(blue), 40,6-diamidino-2-phenylindole

It is well known that the tumor suppressor p53 induces G1 arrest in response to stress. The major downstream effectors of p53 include cyclin D1, Bcl-2 and Bax. Therefore, we further assessed the p53 response after miR-29b-2-5p treatment. As shown in Fig. 5a, miR-29b-2-5p significantly enhanced p53 and p-p53 expression after Cbl-b silencing. Multiple studies showed that p53 ubiquitination and degradation are largely controlled by Mdm2, an E3 ligase. Cbl-b, which is similar to Mdm2, is also an E3 ligase. However, the relationship between Cbl-b and p53 remains undefined. As shown in Fig. 5b, p53 was associated to Cbl-b, with which it could interact (immunoprecipitation, IP) (Fig. 5c). To valuate whether the ubiquitin-proteasome mediated p53 downregulation, the proteasome inhibitor PS341 (5 nM) was incubated for 24 h with SW1990 cells. Interestingly, Cbl-b was associated with p53 in SW1990 cells (Fig. 5d). It is well known that p53 works in the cell nucleus to regulate proliferation. However, it remains unknown p53 is found after Cbl-b inhibition. As expected, miR-29b-2-5p reduced Cbl-b protein expression, while drastically inducing the expression of the nuclear form of p53. Immunofluorescent staining consistently confirmed the induced nuclear p53 expression (Fig. 5e). These findings strongly indicated that miR-29b-2-5p could promote cellular p53 by suppressing Cbl-b, while promoting p53 translocation, from the cytoplasm to the nucleus.

### The expression level of miR-29b-2-5p is negatively correlated with Cbl-b in patients with PDAC

**Fig. 6:**
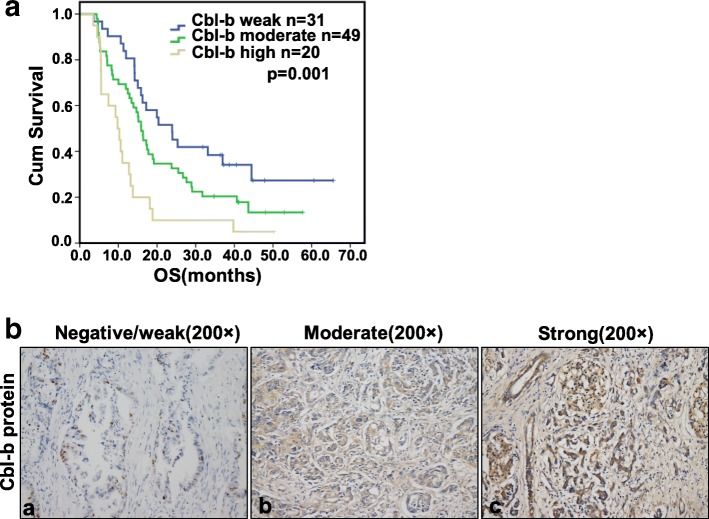
The expression level of Cbl-b protein in tissue samples of 100 patients with PDAC was detected by immunohistochemical method. (**A**) We analyzed the role of Cbl-b in pancreatic cancer, the results showed that the expression level of Cbl-b was significantly negative correlated with the prognosis of pancreatic cancer. (**B**) Immunohistochemical method detect the level of Cbl-b. (**a**) Cbl-b negative staining, (**b**, **c**) Cbl-b moderate and strong staining in cell membrane and cytoplasm (in brown). The original magnification is 200×

The expression levels of the Cbl-b protein in tissue samples from 100 patients with PDAC were detected by immunohistochemistry. We first assessed the role of Cbl-b in pancreatic cancer; interestingly, Cbl-b amounts showed a significant negative correlation with prognosis in pancreatic cancer. Patients with high Cbl-b expression had a median survival of 13.1 months (95% CI 7.9–18.1 months); those with moderate expression had 22.0 months (95% CI 17.1–26.9 months), and the low expression group 32.4 months (95% CI 24.2–40.7 months; *P* = 0.001, Fig. 6A). Furthermore, the pancreatic tumor specimens were grouped according to Cbl-b expression levels as negative/weak, moderate, and strong as determined by immunohistochemical staining (Fig. 6B). The expression level of miR-29b-2-5p was negatively correlated with Cbl-b protein amounts in patients with SPSS (Table 3). Collectively, this clinical and experimental study strongly suggested that Cbl-b promotes PDAC growth.

**Table 3:** The expression level of miR-29b-2-5p was negatively correlated with the expression of Cbl-b protein in patients with SPSS

Cbl-b(*n*,%)	*N*(%)	miR-29b-2-5p(*n*,%)		*R*	*P* value
		Low	High		
Weak	31(31)	10(32)	21(68)	−0.33	0.001
Moderate	49(49)	26(53)	23(47)		
High	20(20)	16(80)	4(20)		
*N*(%)	100(100)	52(52)	48(48)		

## Discussion

In recent years, significant advances in miRNA research have provided clues for understanding the occurrence and development of non-hereditary tumors [32]. Analysis of miRNA expression in clinical follow-up samples has provided valuable information for identifying tumor related prognostic factors [33–35]. However, the molecular regulatory mechanisms of miRNAs in PDAC occurrence and development are rarely studied. In most studies, samples were obtained from PDAC cell lines, PDAC tissues, and normal control tissues [36, 37]. In the present study, patients with similar clinicopathological parameters and treatments but completely different survival outcomes were selected. Among 120 patients with resectable pancreatic cancer, 10 cases with best prognosis and 10 with worst prognosis were selected for miRNA microarray analysis. Then, all cases were verified and a new prognostic model was established. This screening method could be more effective in identifying the potential prognostic values of miRNAs in PDAC.

The miR-29b-2 family has two members, including miR-29b and miR-29b-2-5p [38]. Multiple studies have previously assessed miR-29b as a prognostic factor in many cancers [39]. On the contrary, miR-29b-2-5p is rarely studied. Although miR-29b-2-5p is considered a promoter of bacterial binding to host cells in prokaryotes [40], its identity and function in pancreatic cancer remain unclear. In the current study, miR-29b-2-5p expression independently predicted good survival in PDAC as evaluated by multivariate Cox regression analysis. In addition, miR-29b-2-5p inhibited cell proliferation both in vivo and in vitro, induced cell cycle arrest and promoted apoptosis in pancreatic cell lines. These findings clearly demonstrated for the first time that miR-29b-2-5p was associated with good prognosis and reduced proliferation in PDAC.

It is well known that a single miRNA can modulate multiple cellular signaling pathways by regulating the expression of target genes [41]. The expression and role of Cbl-b in different tissues are very controversial. Previous studies revealed that Cbl-b increases the sensitivity of gastric cancer cells by enhancing the epidermal growth factor receptor (EGFR) and mitochondria mediated signaling pathways in gastric cancer [42]. On the contrary, Cbl-b binds to Smad3 and promotes breast cancer proliferation by inhibiting the TGF-signaling pathway [43]. Our previous study revealed that Cbl-b is regulated by miRNA891b and promote proliferation of PDAC cells by inhibiting the Smad3/p21 pathway [25]. Therefore, the functions of Cbl-b on the proliferation of different cancer cells are absolutely tangled, it may be due to the varied proteins that interact with Cbl-b in different cancer cells.

In this study, the clinical data suggested that pancreatic cancer patients with low miR-29b-2-5p expression and high Cbl-b levels are more likely to have tumor proliferation. Consistently, we demonstrated that Cbl-b overexpression promoted pancreatic cancer cell proliferation both in vitro and in vivo. These findings indicated that Cbl-b is functionally involved in miR-29b-2-5p-mediated tumor growth inhibition in pancreatic cancer cells.

TP53, a classical gene in pancreatic cancer, is associated with apoptosis and G1 phase arrest [44]. Meanwhile, p53 is regulated by MDM2, another E3 ubiquitin ligase. MDM2 inhibits p53 activity in the cytoplasm, promotes p53 degradation and prevents p53 from entering the nucleus and exerts its function [45]. Moreover, previous studies reported that Cbl-b could target Siva 1 and upregulate p53 in lymphoma [46].

However, our results suggested that Cbl-b could bind p53, which in turn is degraded by ubiquitination. More interestingly, Cbl-b inhibition by miR-29b-2-5p resulted in overexpressed p53, which is translocated to the nucleus from the cytoplasm.

## Conclusions

Therefore, the miR-29b-2-5p /Cbl-b/p53 signaling axis provides a basis for further understanding the occurrence and development of PDAC. In summary, miR-29b-2-5p independently predicts better survival in PDAC, as an important tumor suppressor miRNA. Functionally, miR-29b-2-5p inhibits PDAC cell growth by negatively regulating the Cbl-b/p53 axis and reducing Cbl-b-mediated ubiquitination and degradation of p53. These findings provide important clues for understanding the development of PDAC, and suggest miR-29b-2-5p to be a potential biomarker for PDAC prognosis.

## Additional files

Additional file 1: **Figure S2.** The identification of miRNAs. A. The flowchart of miRNA selection and schematic design. B. In the 18 candidate miRNAs, 2 miRNAs were opposite from the miRNA array, 16 were coherent with the miRNA array by Real-time PCR. Good prognosis group/poor prognosis. C. Among the candidate miRNAs, miR-891b and miR-490-5p could inhibit proliferation in cell lines. (PDF 426 kb)

Additional file 2: **Table S1.** Clinical characteristics of the PDAC patients. (DOCX 18 kb)

Additional file 3: **Figure S1.** miRNA-29b-2-5p has a positive correlation with the prognosis of pancreatic cancer by Receiver operating characteristics (ROC) method. A. ROC curves for miR-29b-2-5p indicating the designated cut off points at 0.017. B. miRNA-29b-2-5p has a positive correlation with the prognosis as the cut off value is 0.017 in miRNA validation cohort with a median OS respectively time of 17.8 or 13.7 months. (log rank × 2 = 6.046, *p* = 0.014). (TIF 4419 kb)

Additional file 4: **Figure S3.** Increased expression of miR-29b-2-5p upon infection in 2 PDAC cell lines was confirmed by qRT-PCR. (mean ± SD, results of three independent experiments, *P < 0.05). (PDF 33 kb)

## Abbreviations

Cbl: Casitas B-lineage lymphoma
DAPI: 4′,6-diamidino-2-phenylindole
IP: Immunoprecipitation
miR-29b-2-5p: microRNA-29b-2-5p
PDAC: Pancreatic ductal adenocarcinoma
pN: Pathologic N
pT: Pathologic T
pTNM: Pathologic TNM
TNM: Tumor node metastasis
UPS: Ubiquitin-proteasome system
