# Supplementary figures and images for: MicroRNA-29b-2-5p inhibits cell proliferation by directly targeting Cbl-b in pancreatic ductal adenocarcinoma
Source: BMC Cancer. 2018 Jun 25;18:681. doi: 10.1186/s12885-018-4526-z (PMC6019739; doi:10.1186/s12885-018-4526-z)

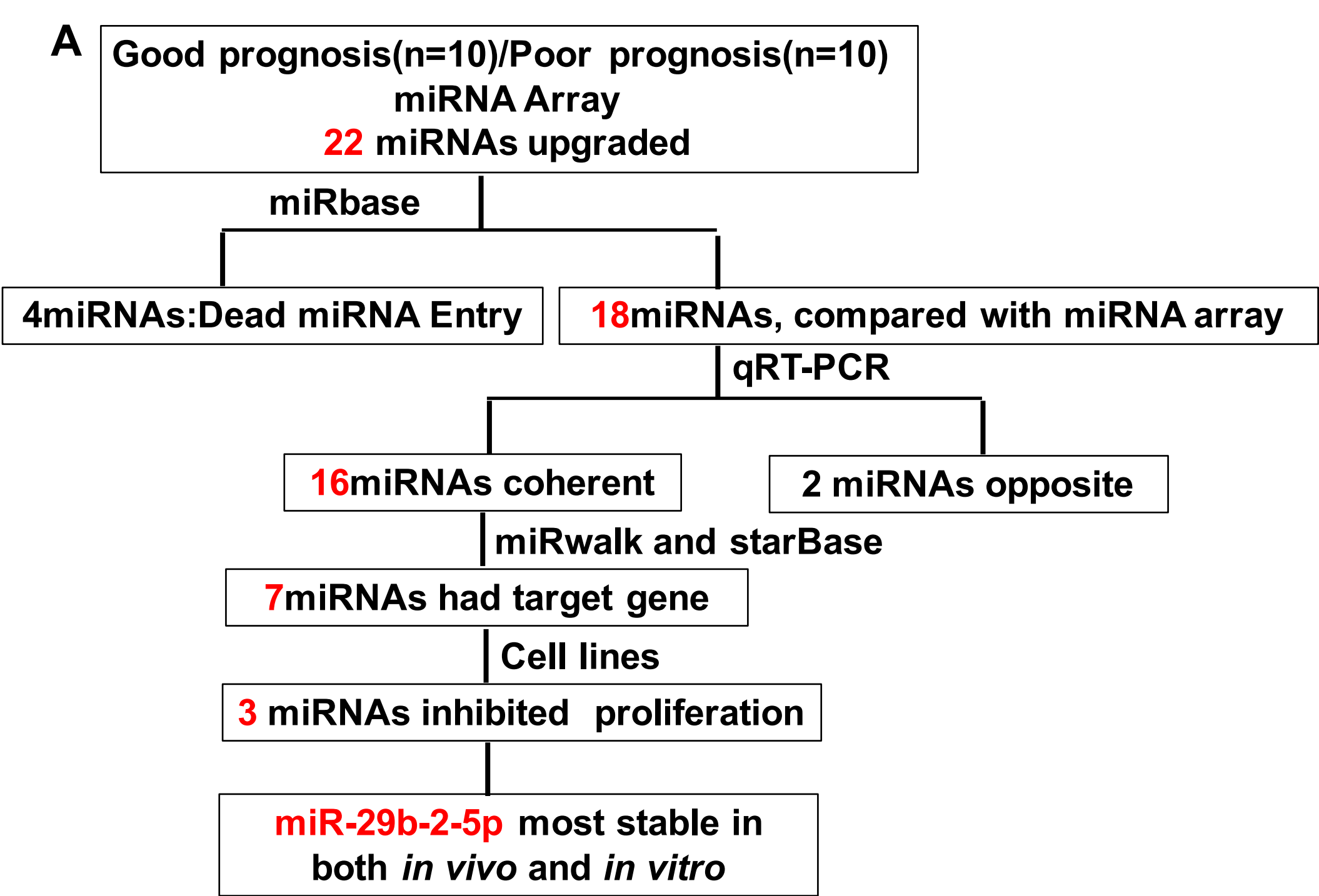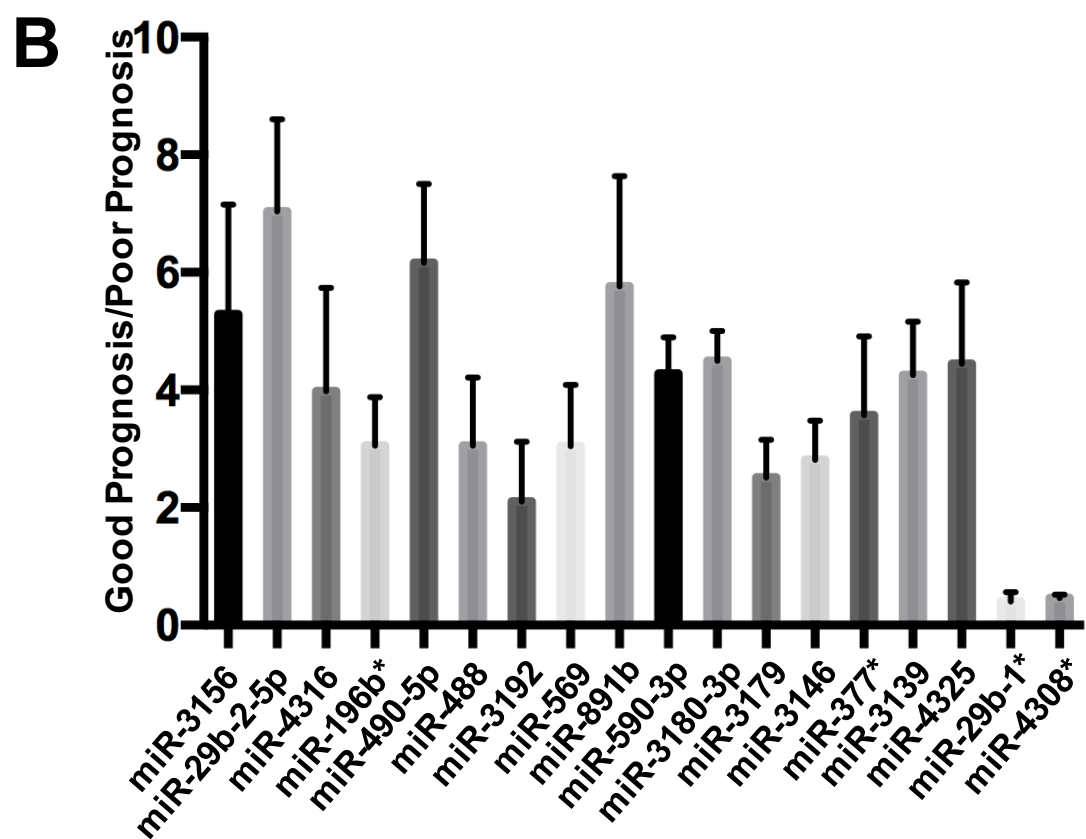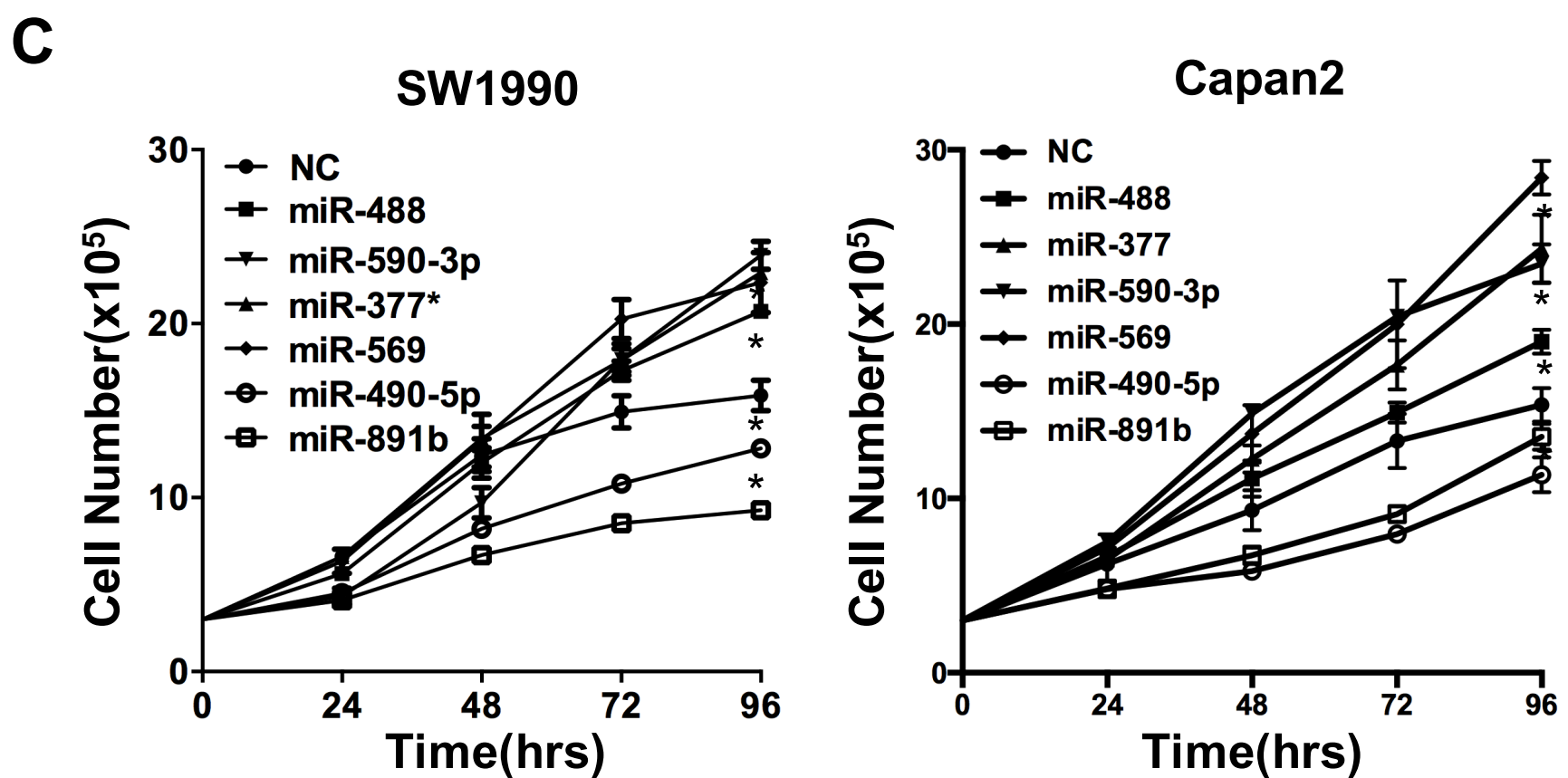

Supplement: Supplementary file 1 — Figure S2. The identification of miRNAs. A. The flowchart of miRNA selection and schematic design. B. In the 18 candidate miRNAs, 2 miRNAs were opposite from the miRNA array, 16 were coherent with the miRNA array by Real-time PCR. Good prognosis group/poor prognosis. C. Among the candidate miRNAs, miR-891b and miR-490-5p could inhibit proliferation in cell lines. (PDF 426 kb) [file 12885_2018_4526_MOESM1_ESM.pdf]

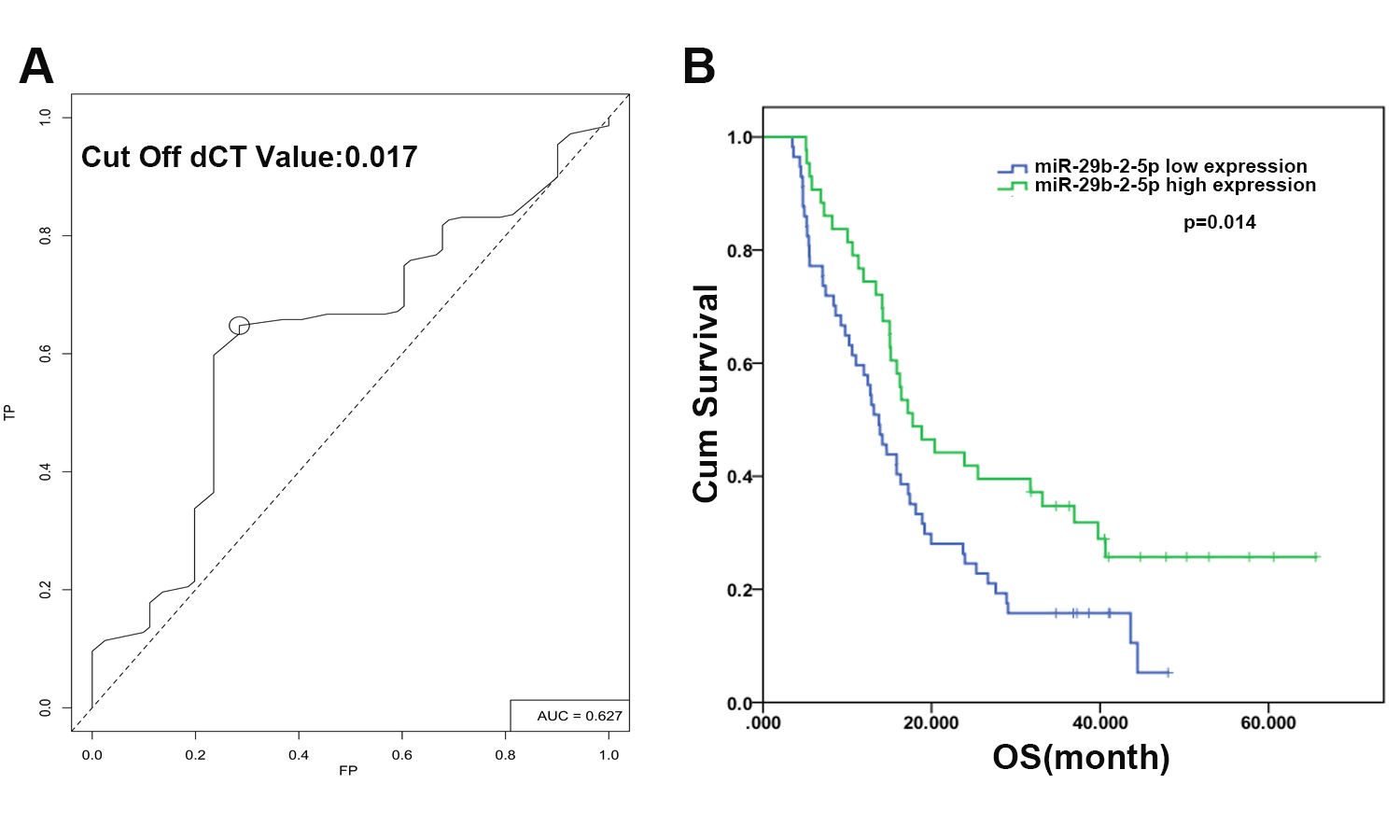

Supplement: Supplementary file 3 — Figure S1. miRNA-29b-2-5p has a positive correlation with the prognosis of pancreatic cancer by Receiver operating characteristics (ROC) method. A. ROC curves for miR-29b-2-5p indicating the designated cut off points at 0.017. B. miRNA-29b-2-5p has a positive correlation with the prognosis as the cut off value is 0.017 in miRNA validation cohort with a median OS respectively time of 17.8 or 13.7 months. (log rank × 2 = 6.046, p = 0.014). (TIF 4419 kb) [file 12885_2018_4526_MOESM3_ESM.tif]

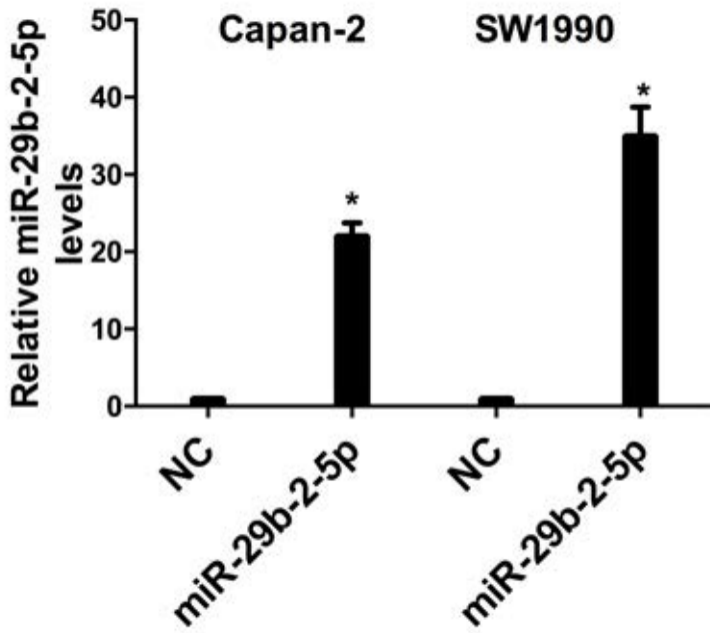

Supplement: Supplementary file 4 — Figure S3. Increased expression of miR-29b-2-5p upon infection in 2 PDAC cell lines was confirmed by qRT-PCR. (mean ± SD, results of three independent experiments, *P < 0.05). (PDF 33 kb) [file 12885_2018_4526_MOESM4_ESM.pdf]
