# Supplementary material for: MicroRNA-29b-2-5p inhibits cell proliferation by directly targeting Cbl-b in pancreatic ductal adenocarcinoma
Source: BMC Cancer. 2018 Jun 25;18:681. doi: 10.1186/s12885-018-4526-z (PMC6019739; doi:10.1186/s12885-018-4526-z)
Supplement: Supplementary file 2 — Table S1. Clinical characteristics of the PDAC patients. (DOCX 18 kb) [file 12885_2018_4526_MOESM2_ESM.docx]

**Table S1.** Clinical characteristics of the PDAC patients.

| Characteristics |  | Patients of the validation cohort (n,%) |
| --- | --- | --- |
| No. Patients |  | 100（100%） |
| Age (years) | < 60 | 48(48%) |
|  | ≥60 | 52(52%) |
| Gender | Male | 61(61%) |
|  | Female | 39(39%) |
| Location of tumor | Head | 41(41%) |
|  | Body or tail | 59(59%) |
| Type of operation | Pancreaticoduodenectomy | 77(77%) |
|  | Distal pancreatectomy | 23(23%) |
|  | Total pancreatectomy | 0 |
| Maximum tumor diameter (cm) | ＜4 | 59(59%) |
|  | ≥4 | 41(41%) |
| Differenciation | Well | 60(60%) |
|  | Moderately | 35(35%) |
|  | Poor | 5(5%) |
| Surgical margins | Negative | 97(97%) |
|  | Positive | 3(3%) |
| pT category | pT1+pT2 | 49(49%) |
|  | pT3+pT4 | 51(51%) |
| pN category | pN0 | 27(27%) |
|  | pN1 | 73(73%) |
| Vessel invasion | No | 51(51%) |
|  | Yes | 49(49%) |
| Vascular tumor thrombus | No | 97(97%) |
|  | Yes | 3(3%) |
| Adjacent organs invasion | No | 73(73%) |
|  | Yes | 27(27%) |
| pTNM category | I | 44(44%) |
|  | IIA | 13(13%) |
|  | IIB | 16(16%) |
|  | III | 27(27%) |
| CA19-9 (median, range) (U/mL) |  | 215.6  (0.60–12076.00) |
